# Ensiling characteristics, *in vitro* digestibility and bacterial community structure of mulberry leaf silage with or without the addition of cellulase, protease, and starch

**DOI:** 10.3389/fpls.2025.1517529

**Published:** 2025-02-18

**Authors:** Guoqiang Zhao, Hao Wu, Yangyuan Li, Zhiyi Huang, Jiajun He, Xiangxue Xie

**Affiliations:** ^1^ Research and Development Center, Guangdong VTR Bio-Tech Co., Ltd., Zhuhai, China; ^2^ College of Animal Science and Technology, China Agricultural University, Beijing, China

**Keywords:** mulberry leaf, additives, fermentation quality, *in vitro* digestibility, microbial community

## Abstract

**Objective:**

This study aimed to investigate the effects of cellulase, protease, and starch on the fermentation quality, *in vitro* digestibility, and microbial community of mulberry leaf silage after 30d of ensiling.

**Methods:**

Mulberry leaves (376 g/kg dry matter (DM)) were ensiled with four experimental treatments: i) CON, no additives; ii) CEL, added cellulase (120 U/g fresh matter [FM]); iii) CPR, added cellulase (120 U/g FM) and protease (50 U/g FM); and iv) CPS, added cellulase (120 U/g FM), protease (50 U/g FM), and starch (2% FM).

**Results:**

All treatments with additives improved fermentation quality, showing higher DM (353 ~ 378 vs. 341 g/kg DM), lactic acid (LA) content (51.6 ~ 64.6 vs. 40.2 g/kg DM), lactic acid bacteria (LAB) counts (7.63 ~ 7.73 vs. 7.49 log10 CFU /g of FM), along with lower pH values (4.29 ~ 4.60 vs. 5.09), and DM losses (124 ~ 130 vs. 134 g/kg DM) compared to the CON group. All the additive treated groups showed higher *in vitro* digestibility of DM (698 ~ 720 vs. 618 g/kg DM), *in vitro* digestibility of NDF (395 ~ 412 vs. 336 g/kg DM), and ADF (277 ~ 298 vs. 232 g/kg DM) than CON. Among all the groups, the CPS group exhibited the highest DM content (378 g/kg DM), LA content (64.6 g/kg DM) and LAB counts (7.73 log_10_ CFU /g of FM), with the lowest pH value (4.29) and DM losses (124 g/kg DM). Additionally, the additive treatments increased abundance of bacteria like Firmicutes and *Enteroccocus*, while reducing Proteobacteria abundance, and resulted in lower diversity and richness of the microbial community. Specifically, CPR and CPS silages showed increased *Pediococcus* and decreased *Enterobacter* compared to CON and CEL, and CPS silage had a relatively high abundance of favorable Bacteroidota. Furthermore, the CPS silage exhibited upregulated genetic functions, energy and lipid metabolism, as well as metabolism of cofactors and vitamins compared to the other groups.

**Conclusion:**

The combined application of cellulase, protease, and starch effectively improved the fermentation quality, *in vitro* digestibility, and microbial community of mulberry leaf silage over the 30-day ensiling period.

## Introduction

With the rapid development of livestock husbandry in China, there is growing interest in exploiting novel forage resources with adequate and high-quality biomass. Given China’s current forage utilization, developing woody forage resources is particularly significant. Mulberry (*Morus alba* L.) leaf, an unconventional protein feed resource, offers high nutritional and ecological value and holds promising prospects for ruminants. It has a high crude protein content (15–28%), comparable to alfalfa hay and 40–50% higher than other leguminous forages, and boasts high livestock digestibility (70–90%). The biomass production of fresh mulberry leaf has been estimated to be approximately 1.82 × 10^7^ t in China (Wang and Luo, 2021). Thus, it could serve as an ideal feed resource for ruminant. However, preserving mulberry leaf presents challenges due to their high moisture content and seasonal biomass harvest, which complicate drying, especially during the rainy season. Ensiling may effectively preserve moist mulberry leaf, maintaining high palatability and nutrient content, and ensuring a year-round supply of green forage biomass for animals. However, naturally ensiled mulberry leaf, without proper management or additives, often undergoes poor fermentation due to its high protein content and relatively low water-soluble carbohydrate (WSC) content.

Numerous studies have shown that high quality silage can be achieved by applying various of additives (Guo et al., 2014; Muck et al., 2018). Of the alternatives, cellulase, protease and starch are three potential suitable additives for better fermentation quality. Cellulase is a widely used additive that can degrade the plant cell wall to release soluble WSC as substrates for lactic acid bacteria (LAB) to produce lactic acid (LA), and can also break down structural carbohydrates, thereby improving silage digestibility (Khota et al., 2018). Protease, another enzymatic additive, has greatly been overlooked due to the assumption that they would result in excessive protein degradation in the rumen, ultimately leading to inefficient nitrogen utilization. However, it has attracted much interests in improvements of forage quality in the past decade. It was reported that the application of proteases that have superior stability and activity under the low pH conditions during ensiling could potentially further enhance starch digestibility thus improve fermentation quality in corn and sorghum silages (Muck et al., 2018; Roseira et al., 2023). In addition, increasing doses of protease application was documented to increase the *in vitro* digestibility of DM, NDF and the disappearance of hemicelluloses and protein of alfalfa hay in a quadratic fashion (Colombatto and Beauchemin, 2009). To date, the effects of protease on the fermentation quality of high-protein forage like mulberry leaves during ensiling remain unknown. Similar to the high-protein forage alfalfa, mulberry leaf is also hypothesized to exhibit beneficial effects when treated with an appropriate dose of exogenous protease. Sugar additives, such as glucose and molasses, have been widely utilized as fermentation stimulants to increase the readily fermentable substrates for LAB activities, and have been shown to increase the DM and LA contents, lower pH value and ammonia nitrogen content in treated silages (Guo et al., 2014; Mu et al., 2021). Similarly, starch, as an exogenous substrate source, has been proven to decrease pH value, increase LA and lactic acid to acetic acid ratio (LA/AA), thereby demonstrating beneficial effects in Napier grass silage (Zhao et al., 2021a). However, its feasibility for mulberry leaf silage remains uncertain, as the effects of additives may vary with different crops.

In recent years, the rapid development of next-generation sequencing (NGS) technology has provided deep understanding of the bacterial communities during silage fermentation process and it is now extensively recognized that the structure of bacterial communities and the abundance of dominant bacteria are key factors affecting the fermentation quality of silage (Guan et al., 2020). Previous studies have indicated that high quality silages are dominated by high abundance of beneficial bacteria like *Lactobacillus*, and low abundance of harmful bacteria such as *Clostridium* or *Enterobacter* (Cheng et al., 2022b). However, the majority of studies have primarily focused on silages treated by LAB inoculants or fibrolytic enzyme additives (Yan et al., 2019; Cheng et al., 2022a). There is limited information available regarding the changes in bacterial community structure and the dynamics of community shifts in silage when treated by cellulase, protease, and starch additives.

Above all, it is hypothesized that additives (cellulase, protease, and starch) would improve the fermentation quality, *in vitro* digestibility and microbial community of mulberry leaf silage. Therefore, the objective of this study was to investigate the ensiling characteristics, *in vitro* digestibility and bacterial community structure of mulberry leaf silage with or without the addition of cellulase, protease, and starch.

## Materials and methods

### Silage preparation

Mulberry leaves (*Yuesang 11*) were harvested in May 2022 at mature leaf stage in the experimental field of Guangdong VTR Bio-Tech Co., Ltd., Zhuhai, Guangdong, China (22°8′19″ N, 113°14′6″ E; elevation -7m; annual mean temperature 22.5°C; precipitation 2,061.9 mm; humidity 79%). The leaves were wilted and chopped into 2–3 cm pieces using a hand cutter. The chopped material was then subjected to one of four treatments: i) no additive control (CON), ii) 120 U/g cellulase (CEL, fresh matter [FM] basis), iii) 120 U/g cellulase and 50 U/g protease (CPR, FM), and iv) 120 U/g cellulase, 50 U/g protease, and 2% starch (CPS, FM). The additives were applied according to the dosages recommended by previous studies with a modification (He et al., 2019b; Der Bedrosian and Kung, 2019; Zhao et al., 2021a). The cellulase and protease were provided by Guangdong VTR Bio-Tech Co., Ltd, while the starch was purchased from Guangzhou Chemical Reagent Factory (Guangzhou, China). The additives were homogenized evenly with 10 ml of distilled water and sprayed on approximately 150 g of fresh mulberry leaves, with an equivalent volume of distilled water applied to the control. The treated materials were mixed thoroughly, ensiled into lab-scale polyethylene bags (28 × 35 cm), and sealed with a vacuum packing machine, with triplicates per treatment. All 12 bags (4 treatments × 3 replicates) were stored at room temperature for 30 days. Each bag was weighed before and after fermentation to determine dry matter (DM) loss. After 30 days, the silage bags were opened, and the contents were mixed thoroughly before sampling. Two subsamples were collected: one for chemical composition and *in vitro* digestibility analysis, and the other for fermentation characteristics and microbial community analysis.

### Chemical composition

Approximately 80 g samples were dried at 65°C for 48 hr in an air-forced drying oven to a constant weight to determine the dry matter (DM) content. The dried samples were then ground using a hand mill to pass through a 1-mm screen, and subsequently used for chemical composition analysis. Total nitrogen (TN) content was measured using the Kjeldahl method according to the Association of Official Analytical Chemists guidelines (AOAC, 2012). Crude protein (CP) content was calculated as TN multiplied by 6.25. True protein nitrogen (TPN) and non-protein nitrogen (NPN) content were analyzed using the method described by Licitra et al. (1996). Briefly, samples (0.5 g) were homogenized with 50 ml distilled water for 30 minutes and sedimented by 10 ml of 10% trichloroacetic acid for 20 to 30 minutes. Subsequently, the mixture was filtered using Whatman No. 54 filter paper (Avantor, USA), and the paper was washed twice with trichloroacetic acid solution. Then the washed paper was dried in a 65°C oven and transferred to determine the TPN content using the Kjeldahl method. NPN was calculated by subtracting the TPN from the TN. The free amino acid nitrogen (FAA-N) proportion was determined using an Assay Kit (Nanjing Jiancheng Bioengineering Institute, Nanjing, China), following the provided manual. Neutral detergent fiber (NDF) and acid detergent fiber (ADF) contents were analyzed using the Van Soest method (Van Soest et al., 1991). The WSC content was quantified using the anthrone-sulphuric acid method (Yemm and Willis, 1954).

### Fermentation characteristics

Fresh silage samples (10 g) were homogenized with 90 ml distilled water at 4°C for 24 hr, as described by He et al. (2018), then filtered through cheesecloth and qualitative filter paper (Whatman No. 6, Avantor, USA). The filtrate was used to determine pH, organic acids, and ammonia nitrogen (NH_3_-N) content. The pH was measured with an electrode pH meter (PHS-3C; Inesa Instrument, Shanghai, China). Organic acids, including lactic acid (LA), acetic acid (AA), and butyric acid (BA) were quantified using a high-performance liquid chromatography (HPLC) system (Agilent 1260, Agilent Technologies, USA) as described by Zhao et al. (2021b). In brief, a portion of filtrate (1.5 ml) was centrifuged at 4,000 rpm for 15 minutes using a centrifuge (Cence H1750, Changsha, China) and the supernatant (700 μl) was filtered with a 1 ml syringe (Double-Dove, Shanghai, China) through a syringe filter with 0.22 μm membrane (Jinteng, Tianjin, China). The HPLC system was equipped with an Agilent Hi-Plex H column (Agilent Technologies, USA) and a 210 nm UV detector (Sciex API 5000, McKinley Scientific, USA). Other instrumental conditions were set as follows: mobile phase: 5 mM H_2_SO_4_; flow rate: 0.7 ml/min; injection volume: 20 μl; temperature: 55°C; pressure: 4.6 MPa. NH_3_-N content was determined by the phenol-sodium hypochlorite colorimetric method (Broderick and Kang, 1980). For microbial enumeration, another 10g sample was mixed with 90 ml sterilized water and serially diluted ten-fold. The LAB were incubated and counted on De Man, Rogosa and Sharpe agar at 37°C for 48 hr under anaerobic conditions. Mold and yeast were incubated on potato dextrose agar at 28°C under aerobic conditions and enumerated after 48 hr. Yeast was differentiated from mold based on colony appearance and cellular morphology. Coliform bacteria were incubated on violet red bile agar at 37°C under aerobic conditions and counted after 18 to 24 hr. All the microbial incubations and enumerations were performed on 90 mm disposable sterile petri dishes (Biosharp, Hefei, China) using the spread-plate method (Olsen and Bakken, 1987). Microbial populations were expressed as colony-forming units (CFU)/g FM and converted to log_10_ units.

### *In vitro* digestibility determination

First, 0.5 g ground samples were weighed into F57 filter bags (Ankom Technology, Macedon, NY, USA) and heat-sealed prior to transferring to the 120 ml glass bottles. The filter bags were pre-rinsed with acetone and thoroughly air-dried to eliminate the surfactant that inhibits microbial digestion. Fresh rumen fluid from three healthy Simmental cattle was collected via stomach tube sucker before morning feeding and immediately filtered through four layers of cheesecloth. The filtrate was then mixed with buffer solution at a 1:1 (v/v) ratio with continuous bubbling of CO_2_. The reagents contained in the buffer solution (per liter) were as follows: 9.8 g NaHCO_3_, 8.12 g Na_2_HPO_4_, 0.47 g NaCl, 0.57 g KCl, 1.0 g Urea, 0.04 g CaCl_2_, and 0.06 g MgCl_2_. Each 120 ml glass bottle received 70 ml of the mixture, including four blank controls, under anaerobic conditions established by 5-second CO_2_ gas flushing. Bottles were then sealed with butyl rubber stoppers equipped with one-way valves of gas outlets and incubated on a DSHZ-300A rotary incubator (Peiying Instrument, Suzhou, China) at 39°C water bath for 48 hr. After incubation, all filter bags were gently rinsed under tap water, oven-dried at 105°C for 4 hr, and weighed to determine *in vitro* digestibility of DM. The NDF and ADF procedures were subsequently performed for the filter bags. The NDF and ADF content of the residue within the bags were determined and used for the calculation of the *in vitro* NDF digestibility and *in vitro* ADF digestibility, respectively.

### Microbial community analysis

The microbial community analysis was performed as per Zhao et al. (2024). Initially, total DNA from fresh silage was extracted using the cetyltrimethylammonium bromide method. DNA concentration and purity were assessed on 1% agarose gels. The V3-V4 hyper-variable region of 16S rRNA genes was amplified with primers 341F (5’-CCTAYGGGRBGCASCAG-3’) and 806R (5’-GGACTACNNGGGTATCTAAT-3’). The amplicons were subsequently purified, quantified, and subjected to paired-end sequencing on an Illumina NovaSeq platform (Novogene, Beijing, China). Paired-end reads were merged as raw tags using FLASH (V1.2.7, http://ccb.jhu.edu/software/FLASH/) and filtered for quality using QIIME (V1.9.1). Effective tags were clustered using QIIME (V1.9.1, http://qiime.org/scripts/splitlibrariesfastq.html). The effective tags were clustered into operational taxonomic units (OTUs) at 97% sequence similarity. OTU results provided relative abundances of different microbial communities at the phylum and genus levels. The alpha diversity indices (Shannon, Simpson, Chao1, and Goods’ coverage) were computed using QIIME (V1.9.1). Furthermore, beta diversity was assessed through principal coordinates analysis (PCoA) using R software (V2.15.3, R Foundation for Statistical Computing, Vienna, Austria). Functional prediction of microbial communities based on Kyoto Encyclopedia of Genes and Genomes (KEGG) databases was performed using Phylogenetic Investigation of Communities by Reconstruction of Unobserved States (PICRUSt). The sequencing data were deposited in NCBI’s Sequence Read Archive (SRA) with the accession number PRJNA1177912.

### Statistical analysis

All statistical analysis of collected data utilized the general linear model procedure in SPSS Statistics (V23.0; IBM, Armonk, NY, USA). The statistical model for analysis was as follows: Yij = μ + Ti + ϵij, where Yij is observation, μ is the general mean, Ti is the fixed effects of experimental treatments (additives), and ϵij is the residual error. Each polyethylene bag was used as the experimental unit in the model. One-way analysis of variance (ANOVA) assessed differences, followed by Duncan’s test for multiple mean comparisons, with statistical significance set at p < 0.05.

## Results and discussion

### Characteristics of mulberry leaves

**Table 1** presents the chemical composition, protein fractions, *in vitro* digestibility, and microbial population of fresh mulberry leaf before ensiling. The DM content of fresh forage significantly influences fermentation quality. In this study, the DM content of fresh mulberry leaf was 376 g/kg FM, slightly exceeding the optimal range of 300 to 350 g/kg FM for high-quality silage, which could mitigate ensiling deterioration. The CP content was 200 g/kg DM, comparable to values previously reported for mulberry leaf (He et al., 2020a). NDF and ADF contents were 295 and 162 g/kg DM, respectively. With its high CP content similar to alfalfa and low fiber content, mulberry leaf shows potential as a high-quality protein feed alternative for animals. The WSC content in fresh materials, crucial for silage quality, measured 104 g/kg DM in this study, surpassing the theoretical requirement of 60–70 g/kg DM for well-fermented silage (Tao et al., 2020). The TPN and NPN were 837 and 163 g/kg TN, respectively. A higher proportion of TPN reportedly suggests superior nutritional value, as NPN is less efficiently utilized by ruminants compared to TPN. The TPN in this study was slightly lower than that of *Neolamarckia cadamba* leaves (887 g/kg TN) and mulberry leaves (885 g/kg TN) reported previously (He et al., 2019a, 2020a), potentially due to variations in plant species, harvesting stage, or cultivation region. The *in vitro* digestibility of DM was 779 g/kg DM, falling within the typical range of 70–90% (Wang and Luo, 2021). Meanwhile the *in vitro* digestibility of NDF and ADF were 654 g/kg DM and 492 g/kg DM, respectively. For successful ensiling, fresh materials typically require at least 5.00 log_10_ CFU/g FM of epiphytic LAB; below 4.00 log_10_ CFU/g FM, DM loss and NH_3_N content may increase (Oliveira et al., 2017). In this study, the LAB population was 3.94 log_10_ CFU/g FM, with a relatively high level of undesirable coliform bacteria (3.47 log_10_ CFU/g FM), posing challenges for quality fermentation without additives. Mold and yeast were also present but at levels below 3.00 log_10_ CFU/g FM, underscoring the necessity of additives for effective preservation.

**Table 1:** Chemical composition, protein fractions, *in vitro* digestibility, and microbial population of mulberry leaf prior to ensiling (Mean ± SD).

Item	Mulberry leaves
Chemical composition	
DM (g/kg FM)	376 ± 3.30
CP (g/kg DM)	200 ± 3.73
NDF (g/kg DM)	295 ± 8.05
ADF (g/kg DM)	162 ± 4.50
WSC (g/kg DM)	104 ± 2.58
Protein fractions	
TPN (g/kg TN)	837 ± 2.90
NPN (g/kg TN)	163 ± 2.90
FAA-N (g/kg TN)	11.9 ± 0.81
*In vitro* digestibility	
DM (g/kg DM)	779 ± 1.46
NDF (g/kg DM)	654 ± 8.16
ADF (g/kg DM)	492 ± 6.74
Microbial population	
LAB (log_10_ CFU/g FM)	3.94 ± 0.16
Mold (log_10_ CFU/g FM)	< 3.00
Yeast (log_10_ CFU/g FM)	< 3.00
Coliform bacteria (log_10_ CFU/g FM)	3.47 ± 0.25

DM, dry matter; CP, crude protein; NDF, neutral detergent fiber; ADF, acid detergent fiber; WSC, water-soluble carbohydrate; TPN, true protein nitrogen; TN, total nitrogen; NPN, non-protein nitrogen; FAA-N, free amino acid nitrogen; LAB, lactic acid bacteria; CFU, colony-forming unit, FM, fresh matter; SD, standard deviation.

### Chemical composition, nitrogen fractions, and *in vitro* digestibility of mulberry leaf silage

Silages treated with additives (CEL, CPR, and CPS) exhibited significantly higher DM content than CON, with the highest observed in the CPS group (p=0.001) (**Table 2**). Application of cellulase (CEL, CPR, and CPS) reduced NDF content, with CPR and CPS showing lower values compared to other groups (p < 0.001). All additive-treated groups showed markedly decreased ADF content compared to CON (p < 0.001). These changes are attributed to hydrolysis and acid solubilization of structural carbohydrates like cellulose and hemicellulose due to cellulase addition, releasing glucose that serves as substrates for LAB proliferation and growth. These findings align with previous studies noting decreased NDF and ADF in cellulase-applied silages (He et al., 2018; Su et al., 2019). Regarding WSC content, protease addition significantly impacted CPR and CPS silages, which showed higher WSC content than CON and CEL, with CPS demonstrating the highest value (p < 0.001). This effect is likely due to amino acids released by protease activity, which may enter metabolic pathways facilitating the conversion of specific amino acids into intermediates for carbohydrates synthesis (Wu, 2009). However, the detailed mechanism of amino acid metabolism and carbohydrate synthesis during mulberry leaf silage fermentation with protease addition remains unclear and requires further study. Hydrolysis of exogenously added starch by amylase naturally present in mulberry leaf might lead to increased glucose release compared to other groups, resulting in higher residual WSC levels. These results aligned with a previous study that demonstrated elevated levels of WSCs in Napier grass silages with starch addition (Zhao et al., 2021a). The application of protease (CPR and CPS) significantly reduced CP content as expected (p < 0.001), along with TPN proportion. In contrast, NPN proportion was higher in protease-treated silages (CPR and CPS) compared to CON and CEL (p < 0.001). Even with protease addition, the TPN proportion in this study (574 and 575 g/kg TN) remained relatively high compared to previous studies on alfalfa and mulberry leaf silage (350–500 g/kg TN) (Wang et al., 2020; Li et al., 2018b). The increased NPN content with protease treatment is attributed to enhanced deamination of amino acids or peptides during ensiling. Alongside NPN, free amino acid nitrogen (FAA-N) and ammonia nitrogen (NH_3_-N) are critical indicators of proteolysis, where FAA-N reflects peptide bond hydrolysis extent and NH_3_-N specifically indicates deamination of peptides or amino acids. Both indicators, FAA-N and NH_3_-N, reflect the generation and utilization results. For instance, FAA-N is determined by protein hydrolysis intensity and peptide/amino acid deamination. Likewise, NH_3_-N content is influenced by microbial activity and peptide/amino acid levels (He et al., 2019a). In this study, NH_3_-N levels across all groups remained low, meeting silage preservation standards (< 100 g/kg TN) (Kung et al., 2018). Protease addition increased FAA-N content, with CPS showing the highest increase (p < 0.001). The nitrogen fraction analysis indicated that mulberry leaf protein retained high value post-ensiling. Proteolysis during silage production, generally driven by plant proteases and a high pH environment, partially converts true protein into NPN (Li et al., 2018b). Proteins hydrolyzed by intrinsic proteases yield peptides and amino acids, which are further deaminated by microbes like *Clostridium* and *Enterobacter* into ammonia, amides, and amines (Kung et al., 2018). The NPN generated from proteolysis is less efficient in nitrogen retention than true protein in the rumen, impacting silage nutritional value and causing nitrogen loss through animal emissions (He et al., 2020a). Therefore, minimizing proteolysis during ensiling is generally advisable. Despite the potential for protein degradation, the study found acceptable levels based on protein fraction analysis and other fermentation parameters (**Table 3**). *In vitro* digestibility analysis is widely used to assess forage nutritional value. It is a critical parameter influenced by factors like plant species, chemical composition, and rumen fluid bacteria. In this study, *in vitro* digestibility of DM ranged from 618 to 723 g/kg DM, surpassing the findings of Dong et al. (2020), who reported an *in vitro* digestibility of DM below 53%. Variations in these results may stem from different mulberry leaf varieties or harvest stages. All cellulase-treated groups (CEL, CPR, CPS) exhibited significantly higher *in vitro* digestibility of DM than CON (p < 0.001), with CPR showing the highest values. This improvement is attributed to the favorable microbial environment in the rumen fluid during *in vitro* incubation, facilitated by increased sugar release with cellulase application and protein degradation due to protease supplementation. However, there was no significant difference between CPR and CPS silages, suggesting that the additional application of starch did not further enhance rumen microbial activity. This was inconsistent with a previous study on mulberry leaf silage, which reported increased *in vitro* digestibility of DM when molasses was added (Dong et al., 2020). The discrepancy might be attributed to that the cellulase and protease additions was sufficient to achieve optimal microbial activity and high digestibility during incubation in the current study. For *in vitro* digestibility of NDF and ADF, all cellulase-treated groups exhibited significantly higher values than CON (p < 0.05). This suggests that cellulase addition effectively breaks down structural carbohydrates like NDF and ADF, enhancing their degradation by digestive enzymes and rumen microbes during incubation. Interestingly, neither protease nor starch additions showed discernible effects on the *in vitro* digestibility of NDF and ADF in this study, as no significant differences were observed among CEL, CPR, and CPS silages. These findings align with prior research indicating minimal impact of exogenous protease on *in vitro* digestibility of NDF in corn silage (Young et al., 2012).

**Table 2:** Chemical composition, nitrogen fractions, and *in vitro* digestibility of mulberry leaf silage.

Item	CON^1)^	CEL	CPR	CPS	SEM^3)^	p-value
Chemical composition ^2)^						
DM (g/kg FM)	341^c^	353^bc^	356^b^	378^a^	4.30	0.001
NDF (g/kg DM)	318^a^	273^b^	248^c^	255^c^	8.43	<0.001
ADF (g/kg DM)	177^a^	152^b^	146^b^	145^b^	4.04	<0.001
WSC (g/kg DM)	10.0^c^	12.9^c^	20.6^b^	25.5^a^	1.90	<0.001
Nitrogen fractions						
CP (g/kg DM)	215^a^	214^a^	203^b^	201^b^	1.94	<0.001
TPN (g/kg TN)	629^a^	626^a^	574^b^	575^b^	8.16	<0.001
NPN (g/kg TN)	371^b^	374^b^	426^a^	425^a^	8.16	<0.001
NH_3_-N (g/kg TN)	36.2^a^	27.5^b^	19.7^c^	15.1^d^	2.44	<0.001
FAA-N (g/kg TN)	18.8^c^	19.2^c^	24.9^b^	26.0^a^	0.99	<0.001
*In vitro* digestibility						
DM (g/kg DM)	618^c^	698^b^	723^a^	720^ab^	13.2	<0.001
NDF (g/kg DM)	336^b^	395^a^	404^a^	412^a^	11.3	0.040
ADF (g/kg DM)	232^b^	277^a^	298^a^	295^a^	9.28	0.013

^1)^CON, no additives; CEL, added cellulase (120 U/g FM); CPR, added cellulase (120 U/g FM) and protease (50 U/g FM); CPS, added cellulase (120 U/g FM), protease (50 U/g FM) and starch (2% FM).

^2)^DM, dry matter; NDF, neutral detergent fiber; ADF, acid detergent fiber; WSC, water-soluble carbohydrate; CP, crude protein; TPN, true protein nitrogen; TN, total nitrogen; NPN, non-protein nitrogen; FAA-N, free amino acid nitrogen.

^3)^SEM, standard error of means.

**Table 3:** Fermentation characteristics and microbial population of mulberry leaf silage.

Item	CON^1)^	CEL	CPR	CPS	SEM	p-value
Fermentation characteristics^2)^						
pH	5.09^a^	4.60^b^	4.37^c^	4.29^d^	0.10	<0.001
DM loss (g/kg DM)	134^a^	130^b^	126^c^	124^d^	1.12	<0.001
LA (g/kg DM)	40.2^d^	51.6^c^	59.9^b^	64.6^a^	2.83	<0.001
AA (g/kg DM)	21.3^a^	20.9^a^	18.3^b^	14.2^c^	0.91	<0.001
BA (g/kg DM)	ND	ND	ND	ND	–	–
LA/AA	1.89^d^	2.47^c^	3.27^b^	4.56^a^	0.30	<0.001
Microbial population						
LAB (log_10_ CFU/g of FM)	7.49^c^	7.69^ab^	7.63^b^	7.73^a^	0.03	0.001
Mold (log_10_ CFU/g of FM)	< 2.00	< 2.00	< 2.00	< 2.00	–	–
Yeast (log_10_ CFU/g of FM)	< 2.00	< 2.00	< 2.00	< 2.00	–	–
Coliform bacteria (log_10_ CFU/g of FM)	< 2.00	< 2.00	< 2.00	< 2.00	–	–

^1)^CON, no additives; CEL, added cellulase (120 U/g FM); CPR, added cellulase (120 U/g FM) and protease (50 U/g FM); CPS, added cellulase (120 U/g FM), protease (50 U/g FM) and starch (2% FM).

^2)^DM, dry matter; LA, Lactic acid; AA, Acetic acid; BA, butyric acid; LA/AA, lactic acid: acetic acid; LAB, lactic acid bacteria; CFU, colony-forming unit, FM, fresh matter.

### Fermentation quality and microbial population of mulberry leaf silage

Applying additives significantly influenced pH values (p < 0.001), with the pH values of the four groups ordered as follows: CPS < CPR < CEL < CON (**Table 3**). pH serves as a crucial indicator of microbial activity and silage quality. High-moisture silage is typically well-fermented when pH ≤ 4.2 (McDonald et al., 1991), whereas silage with high DM content (≥ 32% FM) should ideally have a pH ≤ 4.53 for effective preservation (Meeske et al., 2002). In this study, CON maintained a high pH level (5.09), followed by CEL (4.60), likely due to lower organic acid production associated with mulberry leaf’s strong antimicrobial properties (He et al., 2020a). These observed effects could also be demonstrated by the lower LAB population in CON group. Moreover, mulberry leaf’s high buffering capacity may have contributed (Dong et al., 2020). Protease addition partially degraded proteins, as reflected in CP and TPN contents (**Table 2**). This degradation possibly compromised the buffering capacity of the ensiling material, thus lowering the pH values, as evidenced by the lower pH values observed in the protease-treated groups. (CPR: 4.37, CPS: 4.29). DM loss during ensiling primarily results from early-stage aerobic activity and additive type via hetero-fermentation (Ávila et al., 2014). It was also reported that the losses associated with silage fermentation are primarily from CO_2_ production. The LAB that utilize glucose via homolactic fermentation produce only lactate, resulting in no dry matter (DM) loss, whereas LAB that ferment glucose through heterolactic fermentation generate 1 mol CO_2_ per mol of glucose, leading to 24% DM loss (Borreani et al., 2018). In the current study, additive application significantly reduced DM loss (p < 0.001), with CON silage exhibiting the highest loss (134 g/kg DM). This result was in accordance with the lowest LA/AA ratio in CON (1.89), indicating that fermentation was shifted from heterolactic to homolactic pathways when additives were applied, thereby decreasing the DM loss during fermentation. During silage fermentation, LA is primarily generated by LAB from WSC consumption. Higher LA levels generally indicate more efficient WSC conversion. In this study, LA content was highest in the CPS-treated group (p < 0.001). AA, produced by heterofermentative LAB and enterobacteria, typically correlates with high DM loss. In contrast to LA trends, CON had the highest AA content, whereas groups with protease (CPR and CPS) showed lower levels than the CON and CEL (p < 0.001). The lower WSC content in CON and CEL may explain their significantly higher LA levels and lower AA levels compared to the CPR and CPS, as LAB species can shift from homofermentative to heterofermentative metabolism under WSC-deficient conditions, metabolizing LA into AA (Parvin and Nishino, 2009; Li et al., 2018a). Butyric acid (BA), an indicator of poor fermentation due to undesirable activities by microorganisms like *Clostridium* (Wang et al., 2019a), was not detected in this study, suggesting inhibition of unfavorable microbial activities by the acidic ensiling environment. The LA/AA ratio is a critical indicator in silage production, reflecting the balance between homolactic and heterolactic fermentations during ensiling. Homolactic fermentation, where LAB converts WSC into LA, preserves forage nutritional value and inhibits harmful microorganisms. In contrast, heterolactic fermentation produces LA, AA, and ethanol, which can reduce nutritional quality and palatability for livestock. A higher LA/AA ratio indicates predominant homolactic fermentation, while a lower ratio suggests more heterolactic activity. In this study, LA/AA ratios increased significantly with the addition of cellulase, protease, and starch additives (p < 0.001), shifting fermentation towards homolactic pathways. LAB are key microbes in silage fermentation under anaerobic conditions, with higher populations correlating with improved silage quality. Additive-treated groups exhibited significantly higher LAB populations compared to CON (p = 0.001), with the CPS silage showing the highest increase. These additives likely enhanced LAB growth by increasing fermentable sugars, derived from cellulose degradation and starch hydrolysis, promoting release of glucose or other available sugars for LAB metabolism. Moreover, protease application can enhance the availability of nitrogen sources for LAB growth by releasing peptides and amino acids. After 30 days of ensiling, the counts of undesirable microbes, including mold, yeast, and coliform bacteria, were reduced to below 2.00 log_10_ CFU/g of FM. This indicates that LAB-dominated fermentation significantly inhibited spoilage microbes through the acidic environment created by lactic acid accumulation. Overall, the combined addition of cellulase, protease, and starch improved the fermentation quality of mulberry leaf silage by increasing LA levels, boosting LAB populations, and reducing pH and AA content.

### Venn analysis and alpha diversity indices of microbial community of mulberry leaf silage

**Figure 1:**
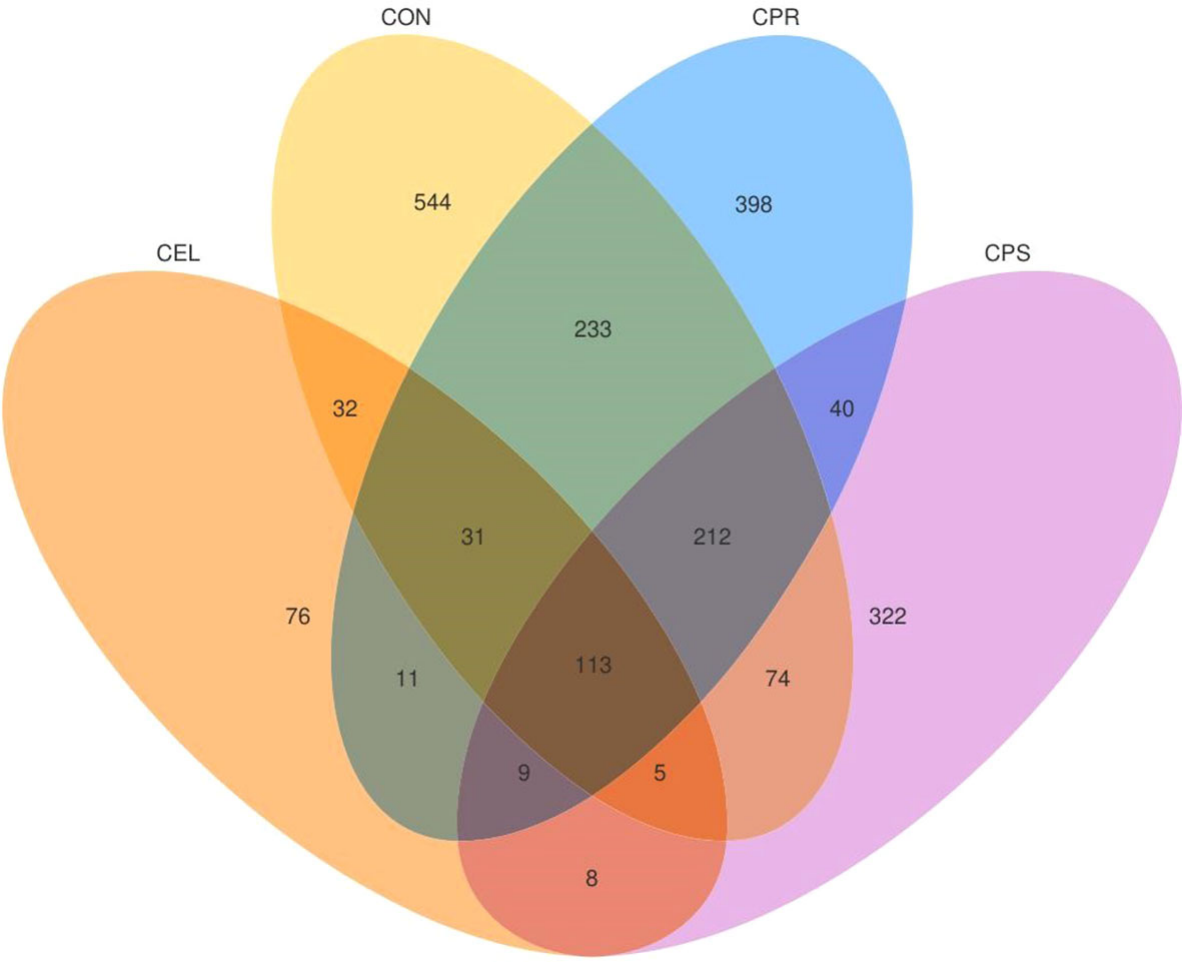
Venn analysis of OTUs in mulberry leaf silage. FM, fresh matter; CON, no additives; CEL, added cellulase (120 U/g FM); CPR, added cellulase (120 U/g FM) and protease (50 U/g FM); CPS, added cellulase (120 U/g FM), protease (50 U/g FM) and starch (2% FM).

Silage fermentation is a complex process influenced by various factors, including the characteristics of the forage, packing density, environmental conditions, and the addition of additives that can affect microbial activity. Additionally, microbial symbiosis, involving numerous microorganisms, plays a crucial role in fermentation and is one of the key determinants of silage quality. Monitoring microbial community changes in silage is essential for understanding the mechanisms and relative abundances of these microorganisms in fermentation. This knowledge can improve silage quality by modifying the microbial communities involved (He et al., 2020b). Venn analysis showed common and specific OTUs among the four silage groups (**Figure 1**). Among the differentially abundant OTUs, 113 were common, while 544, 76, 398, and 322 specific OTUs were found in CON, CEL, CPR, and CPS, respectively. These specific OTUs likely account for the variations in fermentation quality among the different treatment groups. To assess the richness and diversity of microbial communities in the four silage groups, alpha diversity analysis was performed (**Table 4**). High-throughput amplicon sequencing of the 16S rRNA (V3–V4 hypervariable region) yielded a total of 978,264 qualified reads, ranging from 75,501 to 85,802 across all groups. The qualified reads were clustered into OTUs at a 97% similarity level and subjected to microbial community analysis. The Goods’ coverage for all samples exceeded 0.998, indicating sufficiently large sequencing data to accurately profile the silage microbial community (Cheng et al., 2022b). The Shannon and Simpson indices reflect the diversity and evenness of a microbial community, with higher values indicating greater diversity (Yin et al., 2021). The Chao1 index evaluates species richness, with higher values suggesting more species present (Du et al., 2022). In this study, all additive treated groups showed lower Shannon (3.16 ~ 3.66 *vs*. 4.38) and Chao1 indices (198 ~ 370 *vs*. 562) than the CON, while the groups with protease applied (CPR and CPS) exhibited lower Simpson indices (0.699 ~ 0.757 *vs*. 0.883 ~ 0.891) compared to other groups, indicating decreased microbial community diversity and richness due to additives during silage fermentation, and the effects were more substantial to some extent when protease was applied. An increase in dominant bacteria abundance typically corresponds to reduced overall microbial diversity, while a decrease in dominant bacteria correlates with higher diversity (Ogunade et al., 2018). Therefore, it could be inferred that LAB abundance increased with additives, as LAB dominated fermentation in the anaerobic environment and lowered pH through LA production. This acidic environment inhibited undesirable microorganisms, reducing their abundance. Consequently, the microbial communities in additive-treated silages showed lower diversity and richness. These findings were supported by the lower pH, higher LA content, and increased LAB population in the additive-treated groups compared to CON.

**Table 4:** Alpha diversity indices of microbial community in mulberry leaf silage.

Item	CON^1)^	CEL	CPR	CPS	SEM	p-value
Number of reads	75501^c^	85802^a^	84240^ab^	80545^b^	1362	0.006
Goods’ coverage	0.998	0.999	0.998	0.998	0.00	0.532
Observed species	493^a^	151^b^	446^a^	322^ab^	45.9	0.008
Shannon index	4.38^a^	3.55^b^	3.16^b^	3.66^b^	0.15	0.005
Simpson index	0.891^a^	0.883^a^	0.757^ab^	0.699^b^	0.03	0.037
Chao1 richness index	562^a^	198^c^	370^b^	339^b^	42.6	0.001

^1)^CON, no additives; CEL, added cellulase (120 U/g FM); CPR, added cellulase (120 U/g FM) and protease (50 U/g FM); CPS, added cellulase (120 U/g FM), protease (50 U/g FM) and starch (2% FM).

### Relative abundance of microbial community of mulberry leaf silage

**Figure 2:**
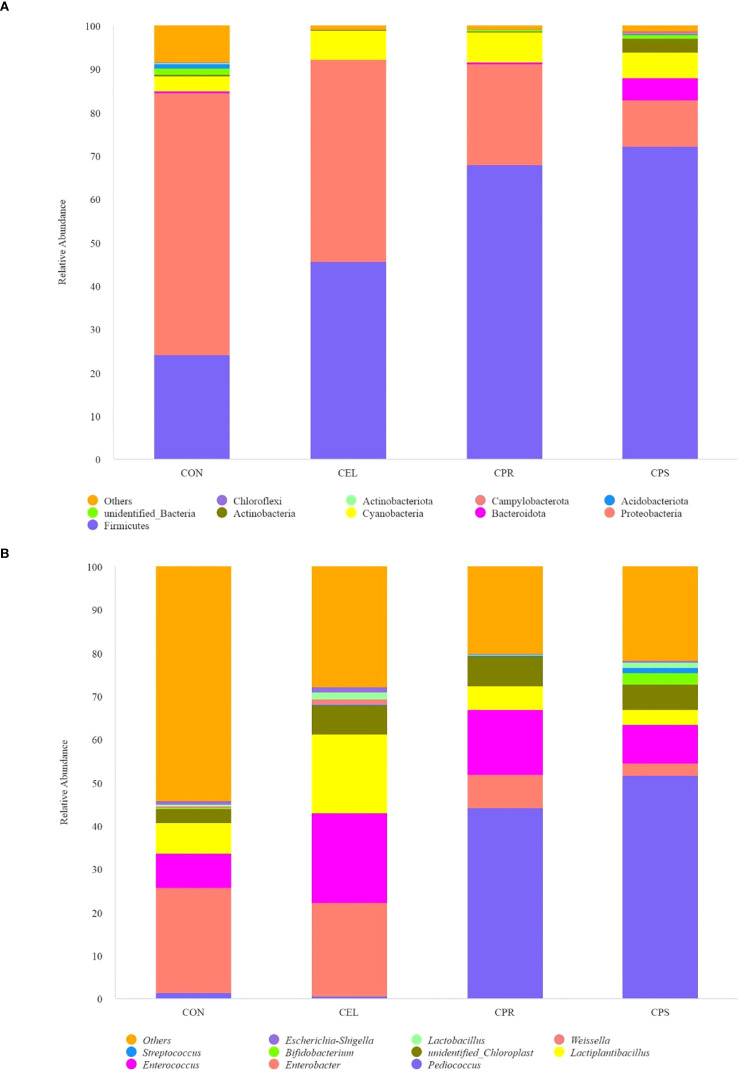
Relative abundance of microbial community on phylum level **(A)** and genus level **(B)** in mulberry leaf silage. CON, no additives; CEL, added cellulase (120 U/g FM); CPR, added cellulase (120 U/g FM) and protease (50 U/g FM); CPS, added cellulase (120 U/g FM), protease (50 U/g FM) and starch (2% FM).

At the phylum level, Firmicutes, Proteobacteria, and Cyanobacteria were the top three dominant phyla, collectively contributing over 85% of the total abundance in all silages (**Figure 2A**). Firmicutes accounted for 24.04% (CON), 45.69% (CEL), 67.90% (CPR), and 72.15% (CPS), while Proteobacteria represented 60.49%, 46.60%, 23.44%, and 10.79% across the four groups, respectively. Cyanobacteria were present at 3.44%, 6.73%, 6.91%, and 5.96% in the respective groups. Firmicutes, essential acid-hydrolytic microorganisms that thrive and secrete various extracellular enzymes under low pH and anaerobic conditions, efficiently degrade complex macromolecules such as cellulose, starch, and protein during ensiling (Romero et al., 2017). The addition of additives in this study significantly increased the abundance of Firmicutes, likely due to the lower pH conditions facilitated by the additives, which promote Firmicutes growth (Keshri et al., 2018). This increase in Firmicutes abundance suggested improvements in silage quality, particularly in the CPR and CPS silages. This finding was consistent with that of Cheng et al. (2022b), who reported that silages treated with additives exhibited a greater abundance of Firmicutes, correlating with higher quality. Proteobacteria, crucial for polysaccharide utilization, organic matter degradation, and carbon and nitrogen cycling during mixed culture fermentation, were also observed (Yuan et al., 2019). Conversely, silages with additives showed a decreased relative abundance of Proteobacteria compared to CON. Cyanobacteria, which typically dominate the microbial community in diverse tropical forages, are epiphytic photosynthesizing bacteria affected by factors such as light, nutrient supply, and temperature (Li et al., 2019). The increased abundance of Cyanobacteria in additive-treated silage may be due to the substrates provided by additives promoting the survival of these microorganisms. Furthermore, the dominant phylum shifted from Proteobacteria in CON to Firmicutes in additive-treated groups, with the Firmicutes/Proteobacteria ratio increasing from 0.40 (CON) to 0.98 (CEL), 2.90 (CPR). and 6.69 (CPS). This shift aligns with previous studies (Wang et al., 2019b; Mu et al., 2020), indicating improved fermentation quality in treated groups. Interestingly, a high abundance of Bacteroidota (5.06%) was observed in CPS silage, while it was below 1% in all other groups. Bacteroidota is known for hydrolyzing complex macromolecular organic matter and releasing enzymes that aid in polysaccharide degradation. Specifically, Bacteroidota play a vital role in the ensiling process by hydrolyzing plant cell wall components, such as cellulose, into monosaccharides, thus improving the digestibility of silage (Yue et al., 2013). Therefore, this suggested that starch addition positively affected the improvement of mulberry leaf silage quality.

Further analysis of the microbial community at the genus level showed that different additive treatments had distinct effects on the microbial community of silages (**Figure 2B**). The top three dominant genera in CON and CEL silages were *Enterobacter* (24.16–21.70%), *Enterococcus* (7.94–20.66%), and *Lactiplantibacillus* (7.09–18.35%). In contrast, the top three genera in CPR silage were *Pediococcus* (44.14%), *Enterococcus* (14.88%), and *Enterobacter* (7.87%), while in CPS silage, they were *Pediococcus* (51.66%), *Enterococcus* (8.95%), and an *unidentified Chloroplast* genus (5.96%). *Enterobacter*, a genus in the phylum Proteobacteria, is commonly found in diverse environments. In silage, *Enterobacter* can have both positive and negative effects on silage quality. Some species can break down complex carbohydrates like plant cell walls and other organic matter, aiding the fermentation process (Ostling and Lindgren, 1995). However, high abundance of *Enterobacter* may result in undesirable effects, as they produce enzymes and acids that contribute to spoilage, decreasing nutritional value and palatability of silage (Li et al., 2019). Additionally, *Enterobacter* is reported to ferment amino acids into NH_3_-N and convert LA to AA and other organic acids (Zi et al., 2021). In this study, additive application decreased *Enterobacter* abundance by 10.18% (CEL), 67.43% (CPR), and 87.87% (CPS) compared to CON. These results were supported by the higher NH_3_-N/TN ratio, increased AA content, and lower LA content in CON silage versus other groups. The relatively high abundance of *Enterobacter* in CON and CEL suggested that ensiling mulberry leaf without additives or with only cellulase is inadequate for high-quality preservation. *Enterococcus*, a genus of Gram-positive, catalase-negative cocci, is commonly found on plant surfaces and in animal intestines (Cai, 1999). As recognized as one of the main lactate-producing bacteria during the ensiling process (Ni et al., 2018), a higher abundance of *Enterococcus* likely leads to increased acidity and a more rapid pH decrease. Similarly, *Pediococcus*, another main LA-producing *cocci*, is extensively found during ensiling. This study found that the exogenous addition of protease dramatically enhanced the abundance of *Pediococcus*, with significantly higher abundance in CPR (44.14%) and CPS (51.66%) compared to CON (1.52%) and CEL (0.58%). These findings may be due to the addition of protease, which provides *Pediococcus* with more readily available nitrogen sources, enhancing its competitiveness against other bacteria. Protease may also specifically benefit *Pediococcus* by improving its metabolic efficiency or growth rate. As a known homofermentative LAB, the predominance of *Pediococcus* in CPR and CPS silages suggests these groups were driven by homo-fermentation, evidenced by higher LA content, a higher LA/AA ratio, and lower AA content. *Lactiplantibacillus*, a rod-shaped LAB, reduces pH during silage fermentation by converting plant carbohydrates into LA (Cheng et al., 2022a).

In this study, *Lactiplantibacillus* was found in relatively high abundance in CEL silage (18.35%), compared to 7.09%, 5.53%, and 3.32% in CON, CPR, and CPS, respectively. It is speculated that the addition of cellulase alone may create a suitable environment for *Lactiplantibacillus* growth. However, when protease or a combination of protease and starch were added with cellulase, the potential interactions among these additives might counteract the beneficial effects. The study observed an abundance of *Unidentified Chloroplast* ranging from 3.40% to 6.91%, but their roles during silage fermentation remain unclear due to limited reports. Overall, the analysis of relative abundance indicated that the microbial community was optimized by the addition of additives, especially the combination of three additives.

### Beta diversity analysis of mulberry leaf silage

**Figure 3:**
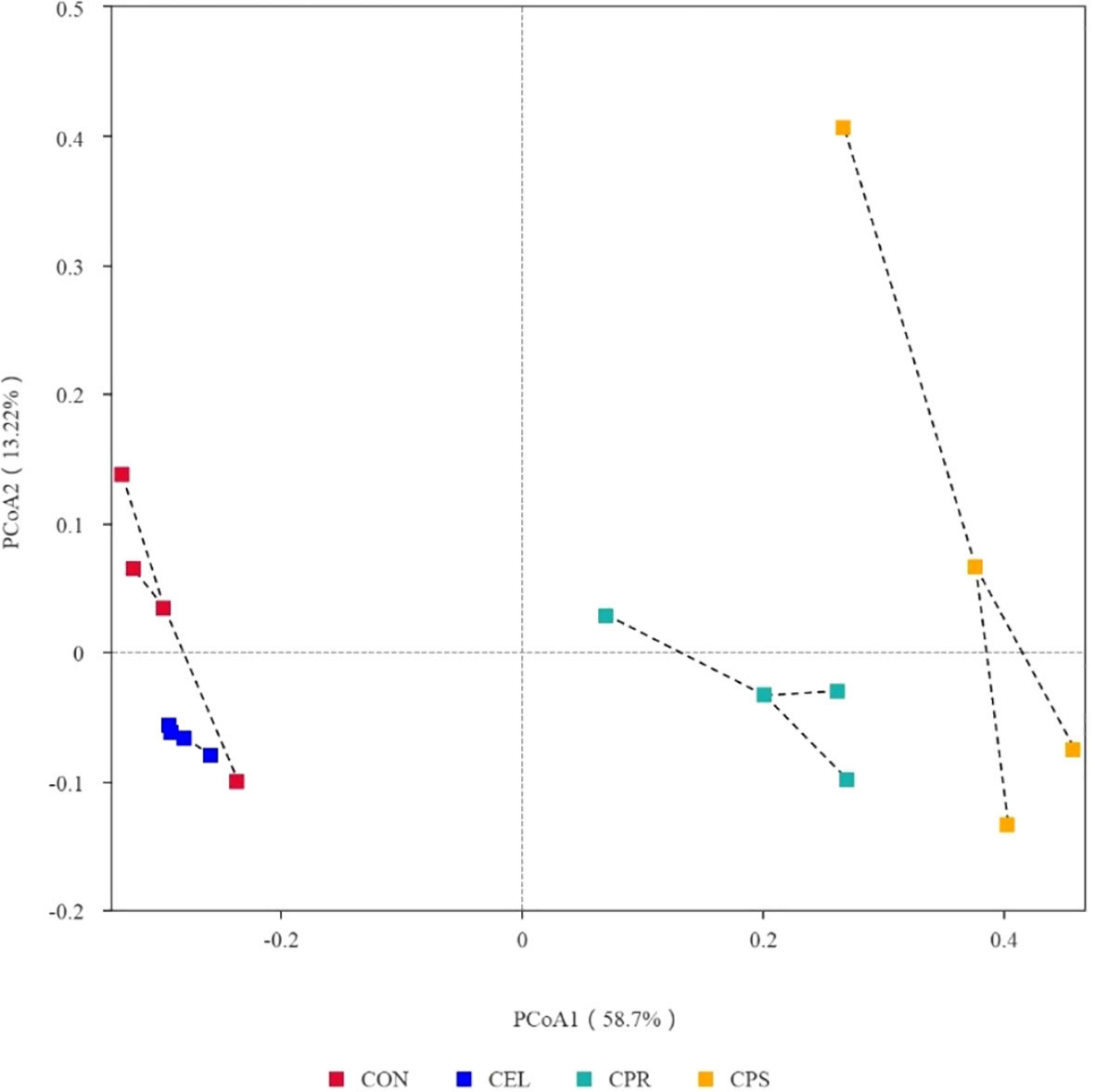
Principal co-ordinates analysis (PCoA) analysis of microbial community in mulberry leaf silage. CON, no additives; CEL, added cellulase (120 U/g FM); CPR, added cellulase (120 U/g FM) and protease (50 U/g FM); CPS, added cellulase (120 U/g FM), protease (50 U/g FM) and starch (2% FM).

To further understand the effects of different additive treatments on microbial community variations, beta-diversity analysis was conducted using PCoA (**Figure 3**). PCoA 1 and PCoA 2 accounted for 58.7% and 13.22% of the total variance, respectively. The silage plots with similar microbial communities clustered together, while those with distinct communities were distributed separately (Parvin et al., 2010). This distinct separation among groups indicates that different additive treatments significantly impacted the microbial communities of the silages. Since microbial community differences can reflect variations in silage quality, it is suggested that applying additives improves fermentation quality by optimizing the microbial community during ensiling.

### Functional prediction of microbial community in mulberry leaf silage

**Figure 4:**
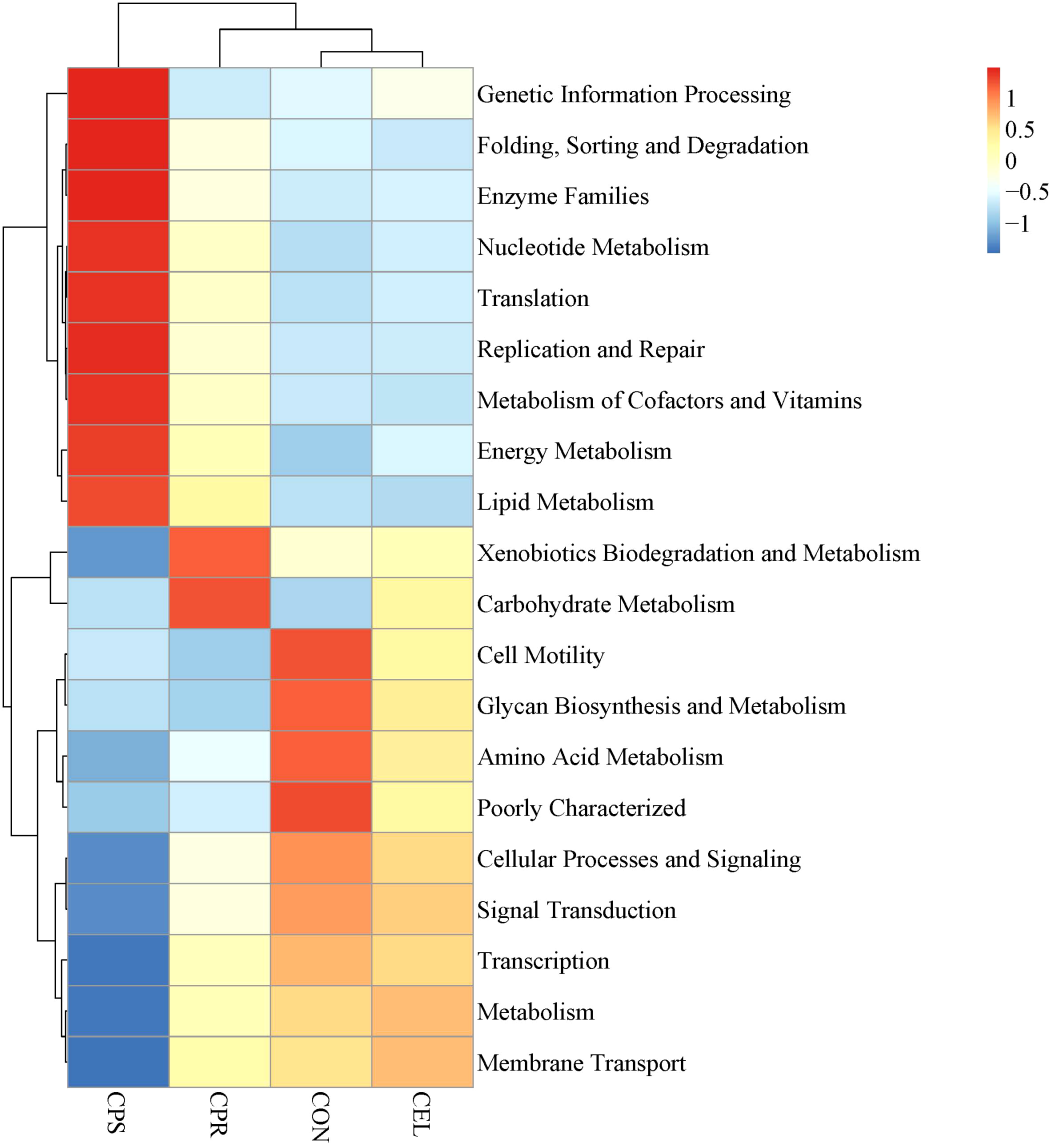
Heatmap of functional prediction of microbial community in mulberry leaf silage. CON, no additives; CEL, added cellulase (120 U/g FM); CPR, added cellulase (120 U/g FM) and protease (50 U/g FM); CPS, added cellulase (120 U/g FM), protease (50 U/g FM) and starch (2% FM).

The heatmap of the top 20 predicted functional pathways at level 2 of microbial community in all silage groups were shown in **Figure 4**. In the current study, the metabolism of carbohydrates, energy, amino acids, genetic functions, and cofactors and vitamins was primarily focused on and compared, as these are closely related to the changes in chemical and fermentation characteristics during ensiling mentioned previously (Bai et al., 2021; Wang et al., 2024b). Compared with the CON, the addition of additives downregulated the amino acid metabolism. Amino acids play a critical role in primary metabolism and the synthesis of bacterial proteins (Jing et al., 2022). Furthermore, LAB do not synthesize all of their essential amino acids, therefore, they require essential amino acids provided by proteolytic systems to support their growth (Bai et al., 2022a). Hence, the different amino acid metabolism might reflect the different dominant microbial communities during fermentation, these were consistent with the results of Bai et al. (2022b), who found that amino acid metabolism was downregulated in high quality silages. The CEL showed upregulated carbohydrate metabolism compared to other groups. The carbohydrate metabolism is one of the most crucial pathways during ensiling process, during which LAB convert WSC into organic acids (Bai et al., 2022a). The promoted carbohydrate metabolism in this study could be a result of cellulase addition, which enhanced the available carbohydrate substrate that could be utilized for LAB activities during ensiling. The CPR treatment enhanced the xenobiotics biodegradation and metabolism, and carbohydrate metabolism. It could be inferred that the addition of cellulase and protease might enhance the activity of bacterial biodegradation to eliminate xenobiotics, which might require the mobilization of metabolism related to carbohydrates and energy. In addition, the CPS group exhibited the upgraded metabolism of genetic information processing, protein folding, sorting and degradation, nucleotide metabolism, translation, DNA replication and repair, metabolism of cofactors and vitamins, and energy and lipid metabolism compared to other groups. It was inferred that the combined application of the three additives could promote the genetic functions including genetic information processing, nucleotide metabolism, translation, replication and repair, folding, sorting and degradation, which might necessitate the mobilization of pathways associated with energy and lipids metabolism. Additionally, these upregulated genetic functions were also likely a result of microorganisms’ responses to prolonged acid stress in silage (Bai et al., 2022a). The promoted energy metabolism in CPS silage was in accordance with the results of Xu et al. (2021), who reported that energy metabolism was enhanced in well fermented silage. It has been reported that some strains of LAB could stimulate the synthesis of vitamins or directly produce vitamins during silage fermentation (Wang et al., 2024a). Therefore, it was deduced that the metabolism of cofactors and vitamins in mulberry leaf silage might be enhanced with the combined application of the cellulase, protease and starch.

## Conclusions

This study demonstrated that mulberry leaf silage fermentation quality can be significantly enhanced after 30 d of ensiling through the addition of additives, especially a combination of three additives. This treatment resulted in higher DM and LA contents, LA/AA ratio, elevated LAB population, enhanced *in vitro* digestibility, and reduced fiber content, pH value, and DM loss compared to CON. Microbial community analysis revealed that all additives boosted the abundance of bacteria such as Firmicutes and *Enterococcus*, while decreasing Proteobacteria abundance, as well as microbial community diversity and richness. Notably, CPR and CPS silages showed increased *Pediococcus* and decreased *Enterobacter* abundance, with CPS silage exhibiting a relatively high abundance of Bacteroidota. The functional prediction of microbial community indicated that the CPS silage exhibited upregulation of genes associated with cellular pathways involved in energy and lipid metabolism as well as metabolism of cofactors and vitamins compared to the other groups. In conclusion, this study recommended the combined application of cellulase, protease, and starch for achieving high-quality mulberry leaf silage preservation.

## Data Availability

The datasets presented in this study can be found in online repositories. The names of the repository/repositories and accession number(s) can be found below: https://www.ncbi.nlm.nih.gov/, PRJNA1177912.

## References

[B1] AOAC (2012). International Official Methods of Analysis (Arlington, VA, USA: AOAC International).

[B2] Ávila C. L. Carvalho B. F. Pinto J. C. Duarte W. F. Schwan R. F. (2014). The use of lactobacillus species as starter cultures for enhancing the quality of sugar cane silage. J. Dairy Sci. 97, 940–951. doi: 10.3168/jds.2013-6987 24359831

[B3] Bai J. Ding Z. Ke W. Xu D. Wang M. Huang W.. (2021). Different lactic acid bacteria and their combinations regulated the fermentation process of ensiled alfalfa: ensiling characteristics, dynamics of bacterial community and their functional shifts. Microb. Biotechnol. 14, 1171–1182. doi: 10.1111/1751-7915.13785 33666350 PMC8085944

[B4] Bai J. Ding Z. Su R. Wang M. Cheng M. Xie D.. (2022a). Storage temperature is more effective than lactic acid bacteria inoculations in manipulating fermentation and bacterial community diversity, co-occurrence and functionality of the whole-plant corn silage. Microbiol. Spectr. 10, E0010122. doi: 10.1128/spectrum.00101-22 35343767 PMC9045155

[B5] Bai J. Franco M. Ding Z. Hao L. Ke W. Wang M.. (2022b). Effect of bacillus amyloliquefaciens and bacillus subtilis on fermentation, dynamics of bacterial community and their functional shifts of whole-plant corn silage. J. Anim. Sci. Biotechnol. 13, 7. doi: 10.1186/s40104-021-00649-0 34991716 PMC8739699

[B6] Borreani G. Tabacco E. Schmidt R. J. Holmes B. J. Muck R. E. (2018). Silage review: factors affecting dry matter and quality losses in silages. J. Dairy Sci. 101, 3952–3979. doi: 10.3168/jds.2017-13837 29685272

[B7] Broderick G. A. Kang J. H. (1980). Automated simultaneous determination of ammonia and total amino acids in ruminal fluid and *in vitro* media. J. Dairy Sci. 63, 64–75. doi: 10.3168/jds.S0022-0302(80)82888-8 7372898

[B8] Cai Y. (1999). Identification and characterization of enterococcus species isolated from forage crops and their influence on silage fermentation. J. Dairy Sci. 82, 2466–2471. doi: 10.3168/jds.S0022-0302(99)75498-6 10575614

[B9] Cheng Q. Chen L. Chen Y. Li P. Chen C. (2022a). Effects of lab inoculants on the fermentation quality, chemical composition, and bacterial community of oat silage on the Qinghai-Tibetan plateau. Microorganisms 10. doi: 10.3390/microorganisms10040787 PMC9026496 35456837

[B10] Cheng Q. Li M. Fan X. Chen Y. Sun H. Xie Y.. (2022b). Effects of epiphytic and exogenous lactic acid bacteria on fermentation quality and microbial community compositions of paper mulberry silage. Front. Microbiol. 13, 973500. doi: 10.3389/fmicb.2022.973500 36090070 PMC9453674

[B11] Colombatto D. Beauchemin K. A. (2009). A protease additive increases fermentation of alfalfa diets by mixed ruminal microorganisms *in vitro* . J. Anim. Sci. 87, 1097–1105. doi: 10.2527/jas.2008-1262 19028863

[B12] Der Bedrosian M. C. Kung L. Jr. (2019). The effect of various doses of an exogenous acid protease on the fermentation and nutritive value of corn silage. J. Dairy Sci. 102, 10925–10933. doi: 10.3168/jds.2019-16436 31563320

[B13] Dong Z. Wang S. Zhao J. Li J. Shao T. (2020). Effects of additives on the fermentation quality, *in vitro* digestibility and aerobic stability of mulberry (Morus alba L.) leaves silage. Asian-Australas J. Anim. Sci. 33, 1292–1300. doi: 10.5713/ajas.19.0420 32054226 PMC7322647

[B14] Du S. You S. Jiang X. Li Y. Wang R. Ge G.. (2022). Evaluating the fermentation characteristics, bacterial community, and predicted functional profiles of native grass ensiled with different additives. Front. Microbiol. 13, 1025536. doi: 10.3389/fmicb.2022.1025536 36329844 PMC9623271

[B15] Guan H. Shuai Y. Yan Y. Ran Q. Wang X. Li D.. (2020). Microbial community and fermentationdynamics of corn silage prepared withheat-resistant lactic acid bacteria in a HotEnvironment. Microorganisms 8. doi: 10.3390/microorganisms8050719 PMC7285033 32408707

[B16] Guo G. Yuan X. Li L. Wen A. Shao T. J. G. S. (2014). Effects of fibrolytic enzymes, molasses and lactic acid bacteria on fermentation quality of mixed silage of corn and hulless–barely straw in the Tibetan plateau. Grassl Sci. 60, 240–246. doi: 10.1111/grs.2014.60.issue-4

[B17] He L. Chen N. Lv H. Wang C. Zhou W. Chen X.. (2020a). Gallic acid influencing fermentation quality, nitrogen distribution and bacterial community of high-moisture mulberry leaves and stylo silage. Bioresour Technol. 295, 122255. doi: 10.1016/j.biortech.2019.122255 31639626

[B18] He L. Lv H. Xing Y. Wang C. You X. Chen X.. (2020b). The nutrients in moringa oleifera leaf contribute to the improvement of stylo and alfalfa silage: fermentation, nutrition and bacterial community. Bioresour Technol. 301, 122733. doi: 10.1016/j.biortech.2020.122733 31935644

[B19] He L. Wang C. Xing Y. Zhou W. Pian R. Yang F.. (2019a). Dynamics of proteolysis, protease activity and bacterial community of neolamarckia cadamba leaves silage and the effects of formic acid and lactobacillus farciminis. Bioresour Technol. 294, 122127. doi: 10.1016/j.biortech.2019.122127 31525585

[B20] He L. Zhou W. Wang Y. Wang C. Chen X. Zhang Q. (2018). Effect of applying lactic acid bacteria and cellulase on the fermentation quality, nutritive value, tannins profile and *in vitro* digestibility of neolamarckia cadamba leaves silage. J. Anim. Physiol. Anim. Nutr. (Berl) 102, 1429–1436. doi: 10.1111/jpn.2018.102.issue-6 30062737

[B21] He L. Zhou W. Wang C. Yang F. Chen X. Zhang Q. (2019b). Effect of cellulase and lactobacillus casei on ensiling characteristics, chemical composition, antioxidant activity, and digestibility of mulberry leaf silage. J. Dairy Sci. 102, 9919–9931. doi: 10.3168/jds.2019-16468 31447142

[B22] Jing Y. Mu C. Wang H. Shen J. Zoetendal E. G. Zhu W. (2022). Amino acid utilization allows intestinal dominance of lactobacillus amylovorus. Isme J. 16, 2491–2502. doi: 10.1038/s41396-022-01287-8 35896730 PMC9561148

[B23] Keshri J. Chen Y. Pinto R. Kroupitski Y. Weinberg Z. G. Sela Saldinger S. (2018). Microbiome dynamics during ensiling of corn with and without lactobacillus plantarum inoculant. Appl. Microbiol. Biotechnol. 102, 4025–4037. doi: 10.1007/s00253-018-8903-y 29536147

[B24] Khota W. Pholsen S. Higgs D. Cai Y. (2018). Comparative analysis of silage fermentation and *in vitro* digestibility of tropical grass prepared with acremonium and tricoderma species producing cellulases. Asian-Australas J. Anim. Sci. 31, 1913–1922. doi: 10.5713/ajas.18.0083 29879827 PMC6212740

[B25] Kung L. Jr. Shaver R. D. Grant R. J. Schmidt R. J. (2018). Silage review: interpretation of chemical, microbial, and organoleptic components of silages. J. Dairy Sci. 101, 4020–4033. doi: 10.3168/jds.2017-13909 29685275

[B26] Li D. Ni K. Zhang Y. Lin Y. Yang F. (2019). Fermentation characteristics, chemical composition and microbial community of tropical forage silage under different temperatures. Asian-Australas J. Anim. Sci. 32, 665–674. doi: 10.5713/ajas.18.0085 30056673 PMC6502719

[B27] Li X. Tian J. Zhang Q. Jiang Y. Wu Z. Yu Z. (2018b). Effects of mixing red clover with alfalfa at different ratios on dynamics of proteolysis and protease activities during ensiling. J. Dairy Sci. 101, 8954–8964. doi: 10.3168/jds.2018-14763 30031582

[B28] Li J. Yuan X. Desta S. T. Dong Z. Mugabe W. Shao T. (2018a). Characterization of enterococcus faecalis jf85 and enterococcus faecium Y83 isolated from Tibetan yak (Bos grunniens) for ensiling pennisetum sinese. Bioresour Technol. 257, 76–83. doi: 10.1016/j.biortech.2018.02.070 29486409

[B29] Licitra G. Hernandez T. M. Van Soest P. J. (1996). Standardization of procedures for nitrogen fractionation of ruminant feeds. Anim. Feed Sci. Technol. 57, 347–358. doi: 10.1016/0377-8401(95)00837-3

[B30] McDonald P. Henderson A. R. Heron S. J. E. (1991). The Biochemistry of Silage. Second Ed (Chalcombe Publications: Marlow, Bucks, UK).

[B31] Meeske R. van der Merwe G. D. Greyling J. F. Cruywagen C. W. (2002). The effect of adding an enzyme containing lactic acid bacterial inoculant to big round bale oat silage on intake, milk production and milk composition of Jersey cows. Anim. Feed Sci. Technol. 97, 159–167. doi: 10.1016/S0377-8401(01)00352-2

[B32] Mu L. Xie Z. Hu L. Chen G. Zhang Z. (2020). Cellulase interacts with lactobacillus plantarum to affect chemical composition, bacterial communities, and aerobic stability in mixed silage of high-moisture amaranth and rice straw. Bioresour Technol. 315, 123772. doi: 10.1016/j.biortech.2020.123772 32653750

[B33] Mu L. Xie Z. Hu L. Chen G. Zhang Z. (2021). Lactobacillus plantarum and molasses alter dynamic chemical composition, microbial community, and aerobic stability of mixed (Amaranth and rice straw) silage. J. Sci. Food Agric. 101, 5225–5235. doi: 10.1002/jsfa.v101.12 33611793

[B34] Muck R. E. Nadeau E. M. G. Mcallister T. A. Contreras-Govea F. E. Santos M. C. Kung L. Jr. (2018). Silage review: recent advances and future uses of silage additives. J. Dairy Sci. 101, 3980–4000. doi: 10.3168/jds.2017-13839 29685273

[B35] Ni K. Zhao J. Zhu B. Su R. Pan Y. Ma J.. (2018). Assessing the fermentation quality and microbial community of the mixed silage of forage soybean with crop corn or sorghum. Bioresour Technol. 265, 563–567. doi: 10.1016/j.biortech.2018.05.097 29861298

[B36] Ogunade I. M. Jiang Y. Pech Cervantes A. A. Kim D. H. Oliveira A. S. Vyas D.. (2018). Bacterial diversity and composition of alfalfa silage as analyzed by illumina miseq sequencing: effects of escherichia coli O157:H7 and silage additives. J. Dairy Sci. 101, 2048–2059. doi: 10.3168/jds.2017-12876 29274960

[B37] Oliveira A. S. Weinberg Z. G. Ogunade I. M. Cervantes A. A. P. Arriola K. G. Jiang Y.. (2017). Meta-analysis of effects of inoculation with homofermentative and facultative heterofermentative lactic acid bacteria on silage fermentation, aerobic stability, and the performance of dairy cows. J. Dairy Sci. 100, 4587–4603. doi: 10.3168/jds.2016-11815 28342607

[B38] Olsen R. A. Bakken L. R. (1987). Viability of soil bacteria: optimization of plate-counting technique and comparison between total counts and plate counts within different size groups. Microb. Ecol. 13, 59–74. doi: 10.1007/BF02014963 24213103

[B39] Ostling C. Lindgren S. (1995). Influences of enterobacteria on the fermentation and aerobic stability of grass silages. Grass Forage Sci. 50, 41–47. doi: 10.1111/j.1365-2494.1995.tb02292.x

[B40] Parvin S. Nishino N. (2009). Bacterial community associated with ensilage process of wilted Guinea grass. J. Appl. Microbiol. 107, 2029–2036. doi: 10.1111/j.1365-2672.2009.04391.x 19548888

[B41] Parvin S. Wang C. Li Y. Nishino N. (2010). Effects of inoculation with lactic acid bacteria on the bacterial communities of italian ryegrass, whole crop maize, Guinea grass and rhodes grass silages. Anim. Feed Sci. Technol. 160, 160–166. doi: 10.1016/j.anifeedsci.2010.07.010

[B42] Romero J. J. Zhao Y. Balseca-Paredes M. A. Tiezzi F. Gutierrez-Rodriguez E. Castillo M. S. (2017). Laboratory silo type and inoculation effects on nutritional composition, fermentation, and bacterial and fungal communities of oat silage. J. Dairy Sci. 100, 1812–1828. doi: 10.3168/jds.2016-11642 28088418

[B43] Roseira J. P. S. Pereira O. G. Da Silveira T. C. Da Silva V. P. Alves W. S. Agarussi M. C. N.. (2023). Effects of exogenous protease addition on fermentation and nutritive value of rehydrated corn and sorghum grains silages. Sci. Rep. 13, 7302. doi: 10.1038/s41598-023-34595-w 37147458 PMC10162983

[B44] Su R. Ni K. Wang T. Yang X. Zhang J. Liu Y.. (2019). Effects of ferulic acid esterase-producing lactobacillus fermentum and cellulase additives on the fermentation quality and microbial community of alfalfa silage. PeerJ 7, E7712. doi: 10.7717/peerj.7712 31608168 PMC6788448

[B45] Tao Y. Sun Q. Li F. Xu C. Cai Y. (2020). Comparative analysis of ensiling characteristics and protein degradation of alfalfa silage prepared with corn or sweet sorghum in semiarid region of inner Mongolia. Anim. Sci. J. 91, E13321. doi: 10.1111/asj.13321 31777177

[B46] Van Soest P. J. Robertson J. B. Lewis B. A. (1991). Methods for dietary fiber, neutral detergent fiber, and nonstarch polysaccharides in relation to animal nutrition. J. Dairy Sci. 74, 3583–3597. doi: 10.3168/jds.S0022-0302(91)78551-2 1660498

[B47] Wang Y. Chen X. Wang C. He L. Zhou W. Yang F.. (2019b). The bacterial community and fermentation quality of mulberry (Morus alba) leaf silage with or without lactobacillus casei and sucrose. Bioresour Technol. 293, 122059. doi: 10.1016/j.biortech.2019.122059 31476563

[B48] Wang S. Ding C. Tian J. Cheng Y. Xu N. Zhang W.. (2024a). An evaluation of storage length on ensiling characteristics, bacterial community compositions, co-occurrence networks, and their functional shifts and pathogenic risk in high-moisture oat silage. Chem. Biol. Technol. Agric. 11. doi: 10.1186/s40538-024-00702-w

[B49] Wang S. Ding C. Tian J. Cheng Y. Xu N. Zhang W.. (2024b). Fermentation profile, bacterial community structure, co-occurrence networks, and their predicted functionality and pathogenic risk in high-moisture italian ryegrass silage. Agriculture 14. doi: 10.3390/agriculture14111921

[B50] Wang C. He L. Xing Y. Zhou W. Yang F. Chen X.. (2019a). Fermentation quality and microbial community of alfalfa and stylo silage mixed with moringa oleifera leaves. Bioresour Technol. 284, 240–247. doi: 10.1016/j.biortech.2019.03.129 30947138

[B51] Wang B. Luo H. (2021). Effects of mulberry leaf silage on antioxidant and immunomodulatory activity and rumen bacterial community of lambs. BMC Microbiol. 21, 250. doi: 10.1186/s12866-021-02311-1 34544373 PMC8454139

[B52] Wang C. Pian R. Chen X. Lv H. Zhou W. Zhang Q. (2020). Beneficial effects of tannic acid on the quality of bacterial communities present in high-moisture mulberry leaf and stylo silage. Front. Microbiol. 11, 586412. doi: 10.3389/fmicb.2020.586412 33224123 PMC7667238

[B53] Wu G. (2009). Amino acids: metabolism, functions, and nutrition. Amino Acids 37, 1–17. doi: 10.1007/s00726-009-0269-0 19301095

[B54] Xu D. Wang N. Rinne M. Ke W. Weinberg Z. G. Da M.. (2021). The bacterial community and metabolome dynamics and their interactions modulate fermentation process of whole crop corn silage prepared with or without inoculants. Microb. Biotechnol. 14, 561–576. doi: 10.1111/1751-7915.13623 32627363 PMC7936295

[B55] Yan Y. Li X. Guan H. Huang L. Ma X. Peng Y.. (2019). Microbial community and fermentation characteristic of italian ryegrass silage prepared with corn stover and lactic acid bacteria. Bioresour Technol. 279, 166–173. doi: 10.1016/j.biortech.2019.01.107 30721817

[B56] Yemm E. W. Willis A. J. (1954). The estimation of carbohydrates in plant extracts by anthrone. Biochem. J. 57, 508–514. doi: 10.1042/bj0570508 13181867 PMC1269789

[B57] Yin X. Tian J. Zhang J. (2021). Effects of re-ensiling on the fermentation quality and microbial community of napier grass (Pennisetum purpureum) silage. J. Sci. Food Agric. 101, 5028–5037. doi: 10.1002/jsfa.v101.12 33570166

[B58] Young K. M. Lim J. M. Der Bedrosian M. C. Kung L. Jr. (2012). Effect of exogenous protease enzymes on the fermentation and nutritive value of corn silage. J. Dairy Sci. 95, 6687–6694. doi: 10.3168/jds.2012-5628 22981573

[B59] Yuan X. Dong Z. Li J. Shao T. (2019). Microbial community dynamics and their contributions to organic acid production during the early stage of the ensiling of napier grass (Pennisetum purpureum). Grass Forage Sci. 75, 37–44. doi: 10.1111/gfs.12455

[B60] Yue Z. Chen R. Yang F. Maclellan J. Marsh T. Liu Y.. (2013). Effects of dairy manure and corn stover co-digestion on anaerobic microbes and corresponding digestion performance. Bioresour Technol. 128, 65–71. doi: 10.1016/j.biortech.2012.10.115 23196223

[B61] Zhao G. Q. Wei S. N. Liu C. Kim H. J. Kim J. G. (2021b). Effect of harvest dates on beta-carotene content and forage quality of rye (Secale cereale L.) silage and hay. J. Anim. Sci. Technol. 63, 354–366. doi: 10.5187/jast.2021.e28 33987610 PMC8071754

[B62] Zhao G. Wu H. Li L. He J. Hu Z. Yang X.. (2021a). Effects of applying cellulase and starch on the fermentation characteristics and microbial communities of napier grass (Pennisetum purpureum schum.) silage. J. Anim. Sci. Technol. 63, 1301–1313. doi: 10.5187/jast.2021.e107 34957445 PMC8672258

[B63] Zhao G. Wu H. Li Y. Li L. He J. Yang X.. (2024). Fermentation characteristics and microbial community composition of wet brewer’s grains and corn stover mixed silage prepared with cellulase and lactic acid bacteria supplementation. Anim. Biosci. 37, 84–94. doi: 10.5713/ab.23.0177 37592379 PMC10766456

[B64] Zi X. Li M. Chen Y. Lv R. Zhou H. Tang J. (2021). Effects of citric acid and lactobacillus plantarum on silage quality and bacterial diversity of king grass silage. Front. Microbiol. 12, 631096. doi: 10.3389/fmicb.2021.631096 33717021 PMC7953137

